# Patient-derived Mammosphere and Xenograft Tumour Initiation Correlates with Progression to Metastasis

**DOI:** 10.1007/s10911-016-9361-8

**Published:** 2016-09-28

**Authors:** Rachel Eyre, Denis G. Alférez, Kath Spence, Mohamed Kamal, Frances L. Shaw, Bruno M. Simões, Angélica Santiago-Gómez, Aida Sarmiento-Castro, Maria Bramley, Mohammed Absar, Zahida Saad, Sumohan Chatterjee, Cliona Kirwan, Ashu Gandhi, Anne C. Armstrong, Andrew M. Wardley, Ciara S. O’Brien, Gillian Farnie, Sacha J. Howell, Robert B. Clarke

**Affiliations:** 1 Breast Biology Group, Breast Cancer Now Research Unit, Division of Molecular and Clinical Cancer Sciences, Manchester Cancer Research Centre, University of Manchester, Wilmslow Road, Manchester, M20 4QL UK; 2 Department of Zoology, Faculty of Science, University of Benha, Benha, Egypt; 3 Pennine Acute Hospitals NHS Trust, Manchester, UK; 4 Salford Royal NHS Foundation Trust, Manchester, UK; 5 University Hospitals of South Manchester NHS Foundation Trust, Manchester, UK; 6 The Christie NHS Foundation Trust, Wilmslow Road, Manchester, M20 4BX UK; 7 Cancer Stem Cell Research, Division of Molecular and Clinical Cancer Sciences, Manchester Cancer Research Centre, University of Manchester, Wilmslow Road, Manchester, M20 4QL UK; 8 Breast Cancer Now Research Unit, Division of Molecular and Clinical Cancer Sciences, Manchester Cancer Research Centre, University of Manchester, Wilmslow Road, Manchester, M20 4QL UK

**Keywords:** Breast cancer, Patient-derived xenografts, Metastasis, Mammosphere, Stem cell activity

## Abstract

Breast cancer specific mortality results from tumour cell dissemination and metastatic colonisation. Identification of the cells and processes responsible for metastasis will enable better prevention and control of metastatic disease, thus reducing relapse and mortality. To better understand these processes, we prospectively collected 307 patient-derived breast cancer samples (*n* = 195 early breast cancers (EBC) and *n* = 112 metastatic samples (MBC)). We assessed colony-forming activity *in vitro* by growing isolated cells in both primary (formation) and secondary (self-renewal) mammosphere culture, and tumour initiating activity *in vivo* through subcutaneous transplantation of fragments or cells into mice. Metastatic samples formed primary mammosphere colonies significantly more frequently than early breast cancers and had significantly higher primary mammosphere colony formation efficiency (0.9 % vs. 0.6 %; *p* < 0.0001). Tumour initiation *in vivo* was significantly higher in metastatic than early breast cancer samples (63 % vs. 38 %, *p* = 0.04). Of 144 breast cancer samples implanted in *vivo*, we established 20 stable patient-derived xenograft (PDX) models at passage 2 or greater. Lung metastases were detected in mice from 14 PDX models. Mammosphere colony formation *in vitro* significantly correlated with the ability of a tumour to metastasise to the lungs *in vivo* (*p* = 0.05), but not with subcutaneous tumour initiation. In summary, the breast cancer stem cell activities of colony formation and tumour initiation are increased in metastatic compared to early samples, and predict metastasis *in vivo*. These results suggest that breast stem cell activity will predict for poor outcome tumours, and therapy targeting this activity will improve outcomes for patients with metastatic disease.

**Electronic supplementary material:**

The online version of this article (doi:10.1007/s10911-016-9361-8) contains supplementary material, which is available to authorized users.

## Introduction

Despite advances in breast cancer diagnoses and treatment, 20 % of patients will develop metastatic tumours at distant sites, eventually leading to death [1]. Metastasis is a multi-step process involving local invasion, intravasation, survival in the circulation, extravasation and colonisation at secondary sites [2]. Although much research has concentrated on understanding the metastatic process, little is known about the cells within a tumour which are able to successfully colonise distant organs. Understanding which cells drive the colonisation and growth of breast cancer cells at distant sites should lead to improvements in adjuvant therapies, designed to prevent the development of secondary tumours.

Much research has focussed on a subset of tumour cells termed cancer stem cells (CSCs), which are capable of self-renewal, and are responsible for tumour initiation [3, 4]. Several methods are used to isolate CSCs and assess their activity, including both functional assays and the expression of cellular markers. Tumour formation following transplantation *in vivo* is considered the gold standard assay to measure CSC activity, and the mammosphere colony forming assay is widely used *in vitro*. This assay was first developed to quantify neural stem cells [5], and was later demonstrated to isolate a stem cell population in mammary tissue [6]. It has since been used to measure cancer stem cell activity in both DCIS and invasive ductal carcinomas [7, 8].

There is evidence that breast cancer cells with a stem cell-like phenotype are metastasis-forming cells in breast cancer. Breast cancer stem-like cells isolated from cell lines are more frequently metastatic compared to non-CSC populations when injected into immunocompromised mice [9, 10]. Furthermore, tumour ALDH1 expression, a marker of breast CSCs, has been shown to be an independent predictive factor for early metastasis in patients [9]. Most recently, single cell analysis of metastatic cells in patient-derived xenograft models revealed that these possessed a stem cell-like gene signature [11]. However the role of CSCs in the metastatic behaviour of patient-derived breast cancer samples is yet to be determined.

Using 307 patient-derived samples from early (*n* = 195, (EBC)) and metastatic (*n* = 112, (MBC)) breast cancers, we evaluated the role of CSC activity in breast cancer metastasis. We assessed both mammosphere colony formation *in vitro* and tumour initiation *in vivo* to determine the relationship between CSC activity and disease progression. Tumour implantation *in vivo* resulted in the production of patient-derived xenograft (PDX) models, and metastasis in these models was correlated to CSC activities in the patient-derived samples. We show that both mammosphere formation *in vitro* and tumour take *in vivo* are increased in metastatic samples compared to early breast cancer samples, and mammosphere formation *in vitro* predicts for metastasis to the lung in PDX models *in vivo*. We thus conclude that cancer stem cell activity *in vitro* correlates with metastasis *in vivo*, and that the mammosphere assay should be further investigated as a tool for identifying metastasis preventing drugs.

## Materials and Methods

### Sample Collection

195 early breast cancer surgical specimens were collected by the Manchester Cancer Research Centre Biobank from patients undergoing surgery for primary tumour removal at University Hospital of South Manchester, Salford Royal and The Pennine Acute Hospitals NHS Trusts. 112 unrelated metastatic samples (pleural effusion or ascitic fluid) were collected from patients during standard therapeutic drainage procedures at The Christie NHS Foundation Trust. All patients underwent fully informed consent as either “basic consent” or “animal consent” in accordance with local research ethics committee guidelines (see Compliance with Ethical Standards section for consenting procedures). Clinical information for samples used in this study (grade, nodal involvement, oestrogen receptor (ER) status, Her2 receptor status, radiotherapy, chemotherapy and endocrine therapy prior to sample collection, Nottingham Prognostic Index (NPI) score) is detailed in Table 1. Cells from samples with basic consent were isolated (see below) and cancer stem cell activity was assessed *in vitro* only. Samples with animal consent were also implanted into mice (see Compliance with Ethical Standards section for animal ethics approvals) to assess tumour initiation *in vivo* (See Supplementary Fig. 1 for sample pathway).

**Table 1:** Summary of clinical data for patients included in the study. 307 patients (195 early and 112 metastatic) were included. A summary of their clinical characteristics is shown. NA; data not available

		**EBC (** ***n*** **= 195)**	**MBC (** ***n*** **= 112)**
Grade	1	8 (4 %)	5 (4.5 %)
	2	83 (42.6 %)	46 (41.1 %)
	3	95 (48.7 %)	34 (30.4 %)
	NA	9 (4.6 %)	27 (24.1 %)
Nodal involvement	Yes	79 (40.5 %)	NA
	No	95 (48.7 %)	NA
	NA	21 (20.7 %)	NA
ER	Pos	133 (68.2 %)	81 (72.3 %)
	Neg	58 (29.7 %)	24 (21.4 %)
	NA	4 (2.1 %)	7 (6.3 %)
Her2	Pos	33 (16.9 %)	10 (8.9 %)
	Neg	148 (75.9 %)	91 (81.3 %)
	NA	14 (7.2 %)	11 (9.8 %)
Radiotherapy	Yes	4 (2.1 %)	NA
	No	191 (97.9 %)	NA
Chemotherapy	Yes	8 (4.1 %)	68 (60.7 %)
	No	187 (95.9 %)	17 (15.2 %)
	NA	0 (0 %)	27 (24.1 %)
Endocrine therapy	Yes	25 (12.8 %)	72 (64.3 %)
	No	170 (87.2 %)	13 (11.6 %)
	NA	0 (0 %)	27 (24.1 %)
NPI	Good (<3.4)	20 (10.3 %)	NA
	Moderate/Poor (>3.4)	152 (77.9 %)	NA
	NA	23 (11.8 %)	NA

### *In vivo* Implantation

120 early breast cancer samples and 24 metastatic breast cancer samples were implanted into female NSG (NOD.Cg-Prkdcscid Il2rgtm1Wjl/SzJ) mice in accordance with the UK Home Office Animals (Scientific Procedures) Act 1986. All tumours were implanted subcutaneously bilaterally into at least two mice per patient sample, either fresh or following one freeze/thaw. Early breast cancers were implanted as 2x2mm^3^ fragments, and metastatic samples were injected as 1 × 10^6^ isolated cancer cells. Metastatic cells were injected in 100 μl of a 50:50 mix of matrigel and mammosphere media (components detailed below). The addition of matrigel to early breast cancer fragments was tested but this did not improve tumour take rate, therefore early breast cancers were implanted without matrigel. The implantation procedure did not change during the duration of the study. Oestrogen supplementation was provided in drinking water for mice with ER positive tumours at a concentration of 8 μg/ml. Tumour growth was measured twice weekly using callipers. When tumours reached 1.3cm^3^ mice were culled and tissue fragments were either implanted into a further generation of mice, frozen for later use, or fixed and examined histologically.

### *In vitro* Sample Processing

Early breast cancers were disaggregated by mincing with a scalpel, prior to digestion in 4.7 ml RPMI medium plus enzymes from the Miltenyi tumour dissociation kit (130–095-929) on a rotating platform at 150 rpm for 2 h. Optimisation was carried out by splitting tumours in half and comparing the following methods: rotating platform vs. gentleMACS tubes, 2 h digestion vs. overnight digestion, Miltenyi tumour digestion enzymes vs. collagenase. In all cases the number of viable cells (assessed using Trypan Blue on a haemocytometer) were counted following digestion. Two hours digestion on a rotating platform with Milteniyi tumour digestion enzymes was found to be optimal. Following digestion, cells were strained through a 70 μM filter (BD, 352340), using a plunger from a 5 ml syringe (Terumo, SS05SE1) to very gently massage the undigested tissue over the filter, and the filter was then rinsed with 3 × 1 ml RPMI medium. Cells were then further strained through a 40 μM filter (BD, 352340), the filter was rinsed with 3 × 1 ml RPMI medium and centrifuged at 1000 g for 5 min at 4 °C to pellet cells. Supernatant was removed and the cell pellet resuspend in 100 μl of ice-cold PBS. Live cells were counted using Trypan Blue (Gibco, 15,250–061) on a haemocytometer, and cells were then cultured as mammospheres (see below). Metastatic samples were first centrifuged at 1000 g for 10 min to pellet cells. Pellets were resuspended in PBS and blood cells were removed by centrifugation of the cell suspension through 0.5 volumes of Lymphoprep solution (Axis Shield, Dundee, UK) at 800 g for 20 min. Epithelial cells were removed from the interface and diluted with PBS before centrifugation at 800 g for 2 min at 4 °C to pellet cells. The supernatant was removed and a cell count performed using Trypan Blue on a haemocytometer. Cells were then cultured as mammospheres (see below).

### Mammosphere Assay

Mammosphere culture was performed as previously described [12]. A single cell suspension was prepared by manual disaggregation (25 gauge needle) and a total of 500 cells/cm^2^ were plated in appropriate polyHEMA (Poly (2-hydroxyethylmethacrylate)) coated tissue culture plates in mammosphere medium (phenol red-free DMEM/F12 (Gibco, 21,041)) containing B27 supplement (no vitamin A; Invitrogen, 12,587), rEGF (20 ng/ml; Sigma, E-9644) and Pen-Strep). Methylcellulose was not added to mammosphere media. Cells were cultured for seven days before mammospheres with diameter greater than 50 μm were counted by visual inspection at ×40 magnification using a microscope fitted with a graticule. Percentage primary mammosphere-forming efficiency (MFE) was calculated by dividing the number of mammospheres formed by the number of cells plated and expressed as a percentage. To assess self-renewal, mammospheres were counted, centrifuged (115×g), and dissociated into a single cell suspension by incubation for 2 min at 37 °C in trypsin EDTA 0.125 % (Sigma), followed by mechanical dissociation (25 gauge needle). Single cells were re-plated at 500 cells/cm^2^ and the number of secondary mammospheres counted after 7 days. Mammosphere self-renewal was calculated by dividing the number of secondary mammospheres formed by the number of primary mammospheres formed.

### Immunohistochemistry

All tumours/cell pellets were fixed in formalin and paraffin embedded. Tumour staining for ER, PR, Ki67 and Her2 was performed in The Christie Hospital Pathology Department. Antibodies used were anti-ERα (Thermo, SP1), anti-PgR (Dako, M3569), anti-Ki67 (Dako M7240) and anti-Her2 (Vector Laboratories, VP-C380). Antigen retrieval was performed either using Target Retrieval Solution pH 9 (Dako S2367, for ER, PR and Ki67) or in 10 mM Citrate buffer (Her2). Antibodies were detected using Dako EnVision Detection System Peroxidase/DAB, Rabbit/Mouse (Dako, K5007) and sections were counterstained with haematoxylin. Hormone receptor positivity was defined as follows; oestrogen receptor (ER) > 5 % positive cells, progesterone receptor (PR) > 5 % positive cells, Her2 receptor 3+ or 2+ confirmed by positive FISH analysis. Human cells were detected in PDX models using a human specific anti-mitochondrial antibody (Abcam, ab92824). Lungs, livers, femurs and lymph nodes were examined for metastases post-mortem in each mouse where subcutaneous tumour growth (P1) was seen. 5 x step sections (40 μM) of each organ were stained with human specific mitochondrial antibody. Sections were stained on a Leica Bond system using standard protocol F and primary antibody conditions of 1:1000 for 15 min. Metastases were detected by visual examination of stained sections. Organs where at ≥1 human cell was observed were classed as metastases positive.

### Statistical Analysis

Statistical analysis was performed using SPSS. Data are represented as mean ± SEM. Statistical significance was measured using parametric testing, assuming equal variance, in the majority of experiments with standard t-tests for two-paired samples used to assess difference between test and control samples. Chi squared analysis was used for categorical variables, The Mann-Whitney U test was used for data which was not normally distributed. Differences were considered statistically significant if the probability value (p) was ≤0.05.

## Results

### Breast Cancer Stem Cell Activity is Increased in Metastatic Breast Cancer Samples

In total, 307 patient-derived samples were collected during this study. These comprised 195 early breast cancers and 112 unrelated metastatic breast cancers. For samples with animal consent, fragments were first removed for implantation before the remainder of the sample was digested for *in vitro* experiments. For samples with basic consent, the whole sample was digested for *in vitro* experiments. Following this sample pathway, 144 samples in total were implanted into mice (120 early breast cancers and 24 metastatic breast cancers). 131 early breast cancer samples and 67 metastatic breast cancer samples were digested for *in vitro* experiments. Of these, 117 early breast cancer samples and 63 metastatic samples yielded viable cells for experiments. The median number of cells isolated from early breast cancer samples was 35,000 and the median number of cells isolated from metastatic breast cancer samples was 5,250,000.

**Fig. 1:**
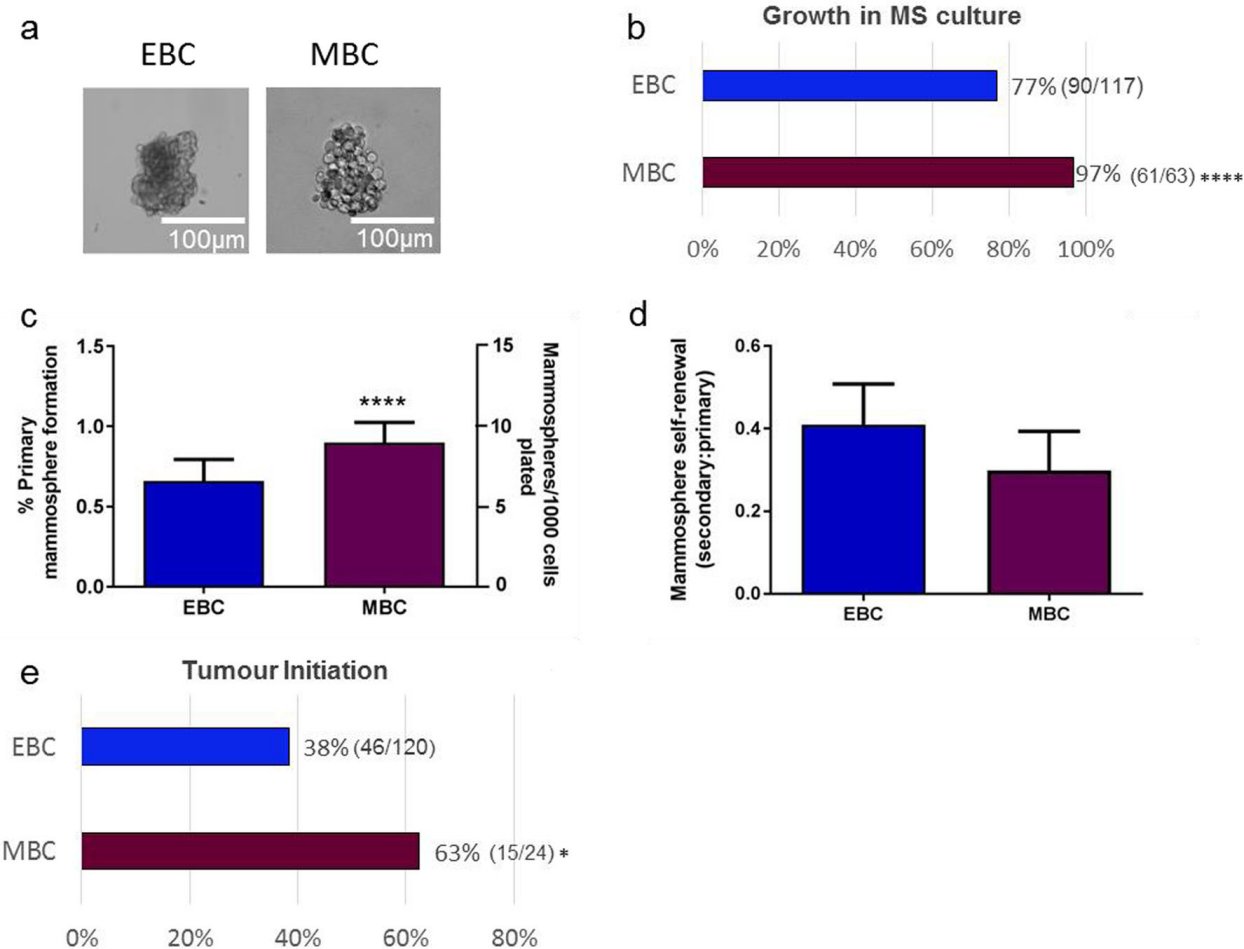
**Breast cancer mammosphere colony formation and** ***in vivo*** **tumour-initiating activity is increased in metastatic compared to early breast cancers**. Cells were isolated from breast cancer samples and grown in suspension culture as mammospheres (representative mammospheres from early (EBC) and metastatic (MBC) samples shown in **a**). Metastatic breast cancer samples were more likely to form mammospheres (MS formation >0) than early breast cancers cultured under the same conditions **b** and had higher primary mammospheres forming efficiencies **c** (Data presented both as % mammosphere formation, and mammospheres formed/1000 cells plated). No difference in secondary mammosphere formation (self-renewal), defined as a ratio of secondary mammospheres: primary mammospheres, was observed between early and metastatic samples **d**. Metastatic samples had significantly higher *in vivo* xenotransplantation potential than early breast cancer samples, over an average period of 200 days, irrespective of tumour phenotype (p = 0.04), where *in vivo* growth is defined as tumour formed to size limit (1.3cm^3^) **e**. Data are presented as mean ± SEM. *p < 0.05 ****p < 0.0005. Statistical analyses: Chi Squared tests (b and e) and two tailed t-test (c and d)

Cancer stem cell activity was assessed using mammosphere colony formation and self-renewal assays in 117 early breast cancers and 63 metastatic breast cancer cases (Representative images of mammospheres in culture Fig. 1a). Patient-derived samples collected represented all molecular subtypes of breast cancer defined by their oestrogen, progesterone and Her2 receptor expression (Clinical information of all samples used in this study is summarised in Table 1). Metastatic tumour cells formed mammospheres (i.e. mammosphere forming efficiency > 0) more frequently than early breast cancers (61/63 (97 %) MBC vs. 90/117 (77 %) EBC; *p* = 0.0003) (Fig. 1b), and had higher mammosphere formation efficiency (0.9 % MBC vs. 0.6 % EBC; *p* < 0.0001) (Fig. 1c). Mammosphere self-renewal rates (measured via secondary mammosphere formation) were similar for early and metastatic breast cancers (0.41 % EBC vs. 0.29 % MBC, *p* = 0.074) (Fig. 1d). Tumour initiation *in vivo* was more frequent in metastatic than early breast cancer samples, with 15/24 (63 %) metastatic samples forming tumours in mice compared to 46/120 (38 %) early samples (*p* = 0.04) (Fig. 1e).

Next, we assessed the relationship between mammosphere colony formation / tumour initiation and clinical parameters in both early and metastatic samples. A summary of all samples which formed mammospheres *in vitro* or tumours *in vivo* is given in Table 2. Mammosphere formation was higher in metastatic samples from patients with ER negative disease than ER positive disease (*p* = 0.02), but not in early breast cancer samples (Supplementary Fig. 2a, b). No association was seen between mammosphere colony formation and tumour grade or molecular subtype for early or metastatic breast cancers (Supplementary Fig. 2c–f), or between mammosphere colony formation and Nottingham Prognostic Index (derived from tumour size, number of involved lymph nodes and grade. <3.4 = good prognosis, ≥3.4 = poor prognosis) in early breast cancers (Supplementary Fig. 2g). Neither mammosphere self-renewal nor *in vivo* tumour initiation ability correlated to the above clinical parameters, although the available numbers of cases where these assays were performed were smaller than those examined for primary mammosphere colony formation (self-renewal: *n* = 36 EBC and *n* = 21 MBC, *in vivo* tumour initiation: *n* = 120 EBC and *n* = 24 MBC).

**Table 2:** Summary of samples exhibiting cancer stem cell properties. Clinical data for all samples which formed mammospheres *in vitro* (n = 90 EBC, n = 61 MBC), a tumour in passage 1 *in vivo* (*n* = 46 EBC, *n* = 15 MBC) or a stable PDX model (*n* = 13 EBC, *n* = 7 MBC) is presented. Data is presented as successful growth / number of samples tested for each clinical characteristic. ER; oestrogen receptor, PR; progesterone receptor, Her2; Her2 receptor, NPI; Nottingham Prognostic Index. NA; data not available

		Formed Mammospheres		Formed tumour *in vivo* (P1)		Formed stable PDX model	
		EBC (*n* = 90)	MBC (*n* = 61)	EBC (*n* = 46)	MBC (*n* = 15)	EBC (n = 13)	MBC (n = 7)
Grade	1	3/4 (75 %)	3/3 (100 %)	1/6 (17 %)	NA	NA	NA
	2	34/45 (76 %)	27/28 (96 %)	22/50 (44 %)	8/14 (57 %)	4/22 (18 %)	3/8 (38 %)
	3	48/63 (76 %)	18/18 (100 %)	30/59 (34 %)	4/6 (67 %)	8/20 (40 %)	2/4 (50 %)
	Unknown	5/5 (100 %)	13/14 (93 %)	3/5 (60 %)	3/4 (75 %)	1/4 (25 %)	2/3 (67 %)
Hormone receptor status	ER+Her2-	50/65 (77 %)	46/48 (96 %)	26/71 (36 %)	10/19 (53 %)	2/26 (8 %)	5/8 (63 %)
	ER+Her2+	12/13 (92 %)	3/3 (100 %)	4/11 (36 %)	1/1 (100 %)	3/4 (75 %)	1/1 (100 %)
	ER-Her2+	6/10 (60 %)	1/1 (100 %)	4/9 (44 %)	1/1 (100 %)	1/4 (25 %)	0/1 (0 %)
	ER-Her2-	20/25 (80 %)	8/8 (100 %)	11/26 (42 %)	3/3 (100 %)	7/11 (64 %)	1/3 (33 %)
	Unknown	2/4 (50 %)	3/3 (100 %)	1/4 (25 %)	0/0 (0 %)	0/1 (0 %)	0/2 (0 %)
NPI	Good (<3.4)	10/12 (83 %)	NA	3/13 (23 %)	NA	0/2 (0 %)	NA
	Moderate/Poor (>3.4)	72/95 (76 %)	NA	33/91 (36 %)	NA	8/22 (36 %)	NA
	NA	8/10 (80 %)	NA	10/16 (63 %)	NA	5/22 (23)	NA

90 breast cancer samples were both implanted *in vivo* and grown as primary mammospheres *in vitro* to assess correlations between *in vivo* and *in vitro* stem cell activity (70 EBC, 20 MBC). Of these, 17 formed tumours when implanted *in vivo* and 53 formed mammospheres *in vitro*. Mammosphere colony formation *in vitro* did not predict tumour initiation *in vivo* (Supplementary Fig. 2h), and there was no difference in the percentage mammosphere formation *in vitro* in samples which were able to initiate tumours *in vivo* (data not shown).

### Patient-Derived Xenografts Recapitulate Clinical Tumours

In total, 144 samples were subcutaneously implanted *in vivo* into NSG mice (120 EBC, 24 MBC). Table 2 provides a summary of all samples implanted, and those which formed a tumour in P1, or a stable PDX model. Of the 144 samples implanted, 61 formed tumours in the first passage (P1) in mice (46 EBC, 15 MBC), and 20 stable PDX tumour models at generation 2 or greater have been established (13 EBC, 7 MBC). These are derived from 7 ER + Her2-, 4 ER + Her2+, 1 ER-Her2+ and 8 ER-Her2- patient samples (Table 3). Of the 13 early breast cancers from which stable PDX lines were derived, 10 patients were treatment naïve, 2 had received endocrine therapy (BB6RC37 and BB6RC160, both treated with Arimidex) and treatment information was not available for 1 patient (BB2RC08). All 7 of the MBC PDX models were derived from patients who had received prior therapy.

**Table 3:** Summary of Patient-Derived Xenografts created in this study. 20 stable PDX models were created during this study. Receptor status information is presented for clinical tumours and PDX tumours. The highest passage number for each model is presented, as is if PDX models spontaneously metastasise to the lung. EBC; early breast cancer. MBC; metastatic breast cancer. IDC; Invasive Ductal Carcinoma, DCIS; Ductal Carcinoma in situ, NA; data not available, NE; not examined

		Clinical information				PDX information				
Model	Type	Pathology	ER	PR	Her2	ER	PR	Her2	Passage	Lung Mets?
BB2RC08	EBC	NA	Pos	NA	Neg	Pos	Neg	Neg	P4	Yes
BB6RC80	EBC	IDC	Pos	Pos	Neg	Pos	Pos	Neg	P3	NE
BB6RC39	EBC	IDC	Pos	Pos	Pos	Pos	Pos	Pos	P3	Yes
BB6RC87	EBC	IDC/DCIS	Pos	Neg	Pos	NA	NA	NA	P1	NE
BB6RC160	EBC	IDC/DCIS	Pos	Neg	Pos	NA	NA	NA	P2	Yes
BB6RC148	EBC	IDC	Neg	Neg	Pos	NA	NA	NA	P3	Yes
BB6RC37	EBC	IDC	Neg	Neg	Neg	Neg	Neg	Neg	P3	No
BB6RC52	EBC	IDC	Neg	Neg	Neg	NA	NA	NA	P4	Yes
BB6RC69	EBC	IDC/DCIS	Neg	Neg	Neg	Neg	Neg	Neg	P3	No
BB6RC88	EBC	IDC	Neg	Pos	Neg	NA	NA	NA	P2	Yes
BB6RC153	EBC	IDC	Neg	Neg	Neg	Neg	Neg	Neg	P2	Yes
BB6RC191	EBC	NA	Neg	Neg	Neg	Neg	Neg	Neg	P2	NE
BB6RC193	EBC	IDC	Neg	Neg	Neg	NA	NA	NA	P1	No
BB3RC29	MBC	NA	Pos	Neg	Neg	Neg	Neg	Neg	P9	Yes
BB3RC31	MBC	IDC	Pos	Pos	Neg	Pos	Pos	Neg	P8	Yes
BB3RC32	MBC	IDC	Pos	Pos	Neg	Pos	Pos	Neg	P4	Yes
BB3RC50	MBC	IDC	Pos	Neg	Neg	Pos	Neg	Neg	P3	Yes
BB3RC72	MBC	NA	Pos	Pos	Neg	NA	NA	NA	P1	NE
BB3RC71	MBC	IDC	Pos	Pos	Pos	NA	NA	NA	P2	NE
BB3RC84	MBC	NA	Neg	Neg	Neg	Neg	Neg	Neg	P2	No

**Fig. 2:**
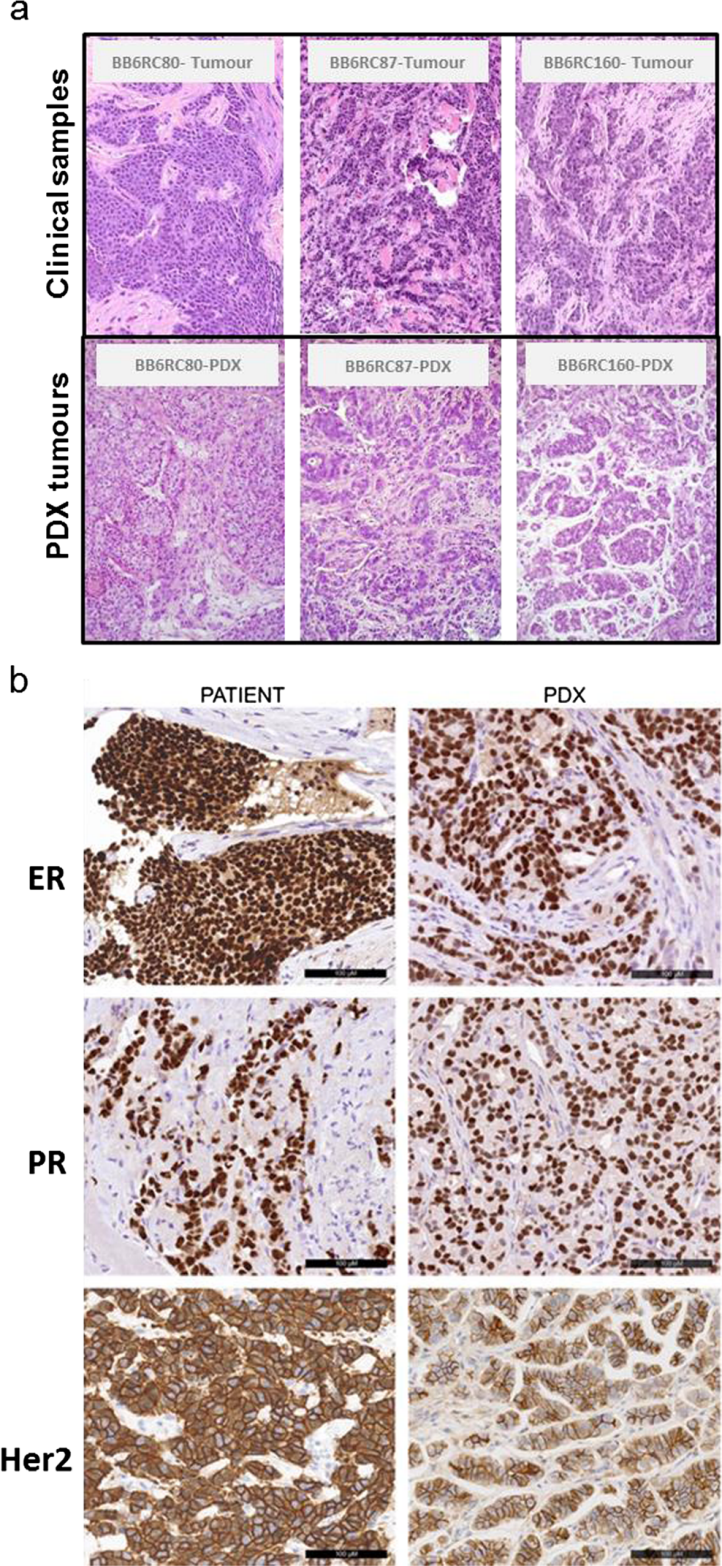
**Characterisation of Patient-Derived Xenograft tumours.** Of the 144 tumours implanted in this study, 61 grew in the first passage in mice and 20 stable PDX models were created. Comparisons of representative patient tumour samples and their corresponding patient-derived xenograft in passage 1 **a**. Patient-derived xenografts retained ER, PR and Her2 status of the patient tumour. Representative images in **b**. Scale bar =100uM

PDX tumours grown in mice resembled patient-derived tumours histologically (Fig. 2a) and retained hormone receptor expression of the patient-derived tumour (Fig. 2b). There was no association between the ability of an early breast cancer sample to form a stable PDX and clinical characteristics (grade, NPI group, ER/PR status, Her2 status) (data not shown).

### Spontaneous Lung Metastasis of PDX Tumour models Correlates with Mammosphere colony Formation

**Fig. 3:**
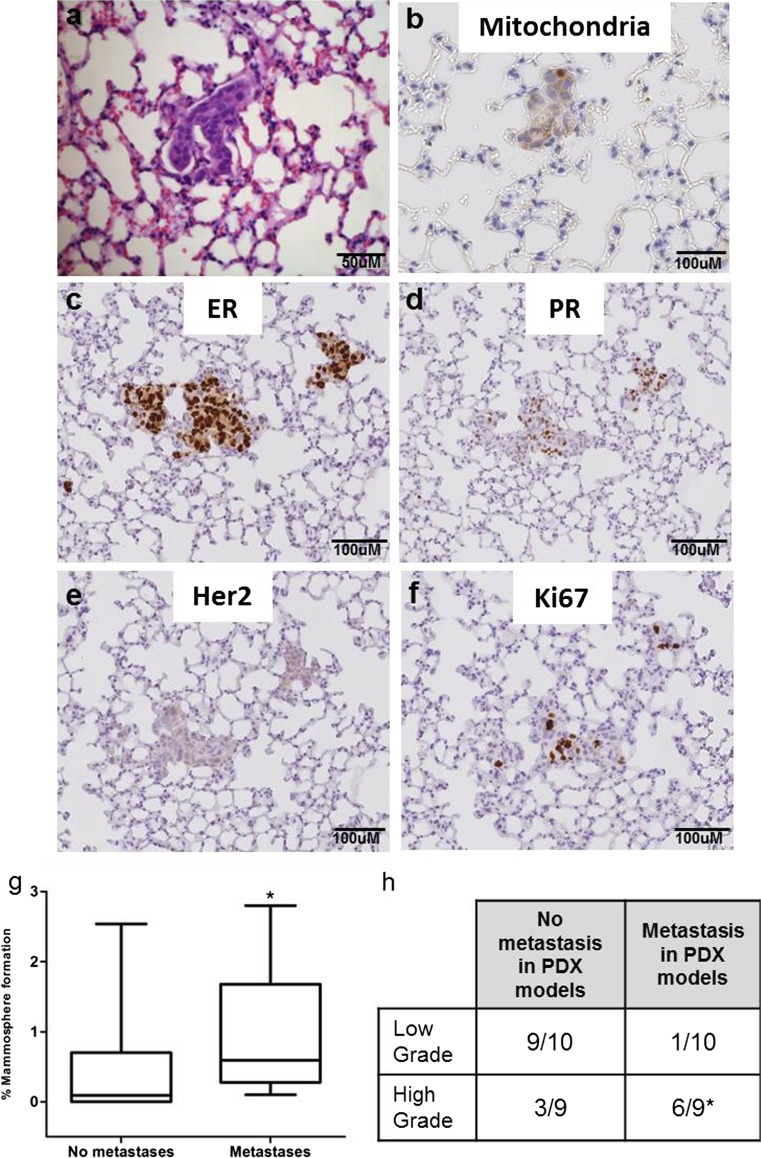
**Mammosphere colony formation predicts metastasis in PDX models.** Lung metastases were detected in 14/34 models where a tumour formed in passage 1 by H&E **a** and confirmed by staining with a human specific mitochondrial antibody **b**. Lung metastases retained the ER **c**, PR **d** and Her2 **e** status of the primary tumour and contained ki67 positive dividing cells **f**. Primary mammosphere formation predicted PDX metastases, with samples which metastasised to the lungs *in vivo* having a significantly higher % mammosphere forming efficiency *in vitro* **g**. Metastases are more likely to form in PDX models from high grade EBC tumours (grade 3) than low grade (grade 1 and 2) **h**. Data are presented as mean ± SEM. *p < 0.05 Statistical analysis: Mann -Whitney U Test

When subcutaneous tumour growth (P1) was seen, the lymph nodes, bones, lungs and livers were examined for the presence of metastases post-mortem. This totalled 34 models, comprising 25 derived from early breast cancers and 9 derived from metastatic breast cancers. Metastatic cells were not observed in the lymph nodes, bones or livers of any of the mice, however lung metastases were detected in mice from 14/34 (41 %) models (representative H&E of lung metastases are shown in Fig. 3a, details of mice with lung metastasis are given in Supplementary Table 1). These included 11 of our 20 stable PDX models (detailed in Table 3). Tumours which metastasised to the lung comprised 7 ER + Her2- models, 3 ER + Her2+ models, 1 ER-Her2+ models and 3 ER-Her2-, from 8 early breast cancer and 6 metastatic samples. Human origin of the cells was confirmed using a human-specific mitochondrial antibody (Fig. 3b). Lung metastases retained the hormone receptor status of the primary tumour (Fig. 3c–e shows ER (Fig. 3c), PR (Fig. 3d) and Her2 (Fig. 3e) from an ER+, PR+, Her2- tumour) and contained Ki67 positive dividing cells (Fig. 3f). Mammosphere colony formation (primary mammosphere generation) by patient-derived tumour cells before implantation into mice predicted the ability of a PDX tumour to metastasise *in vivo*, with samples with a higher percentage mammosphere forming efficiency being significantly more likely to metastasis to the lungs in PDX models (*p* = 0.05) (Fig. 3g).

Finally we investigated whether any clinical parameters correlated to the ability to metastasise *in vivo*. PDX tumours established from high grade, early breast cancer samples were more likely to metastasise to the lungs, with 6/9 high grade (Grade 3) tumours metastasising compared to 1/10 low grade tumours (Grade 1 or 2) (*p* = 0.02) (Fig. 3h).

## Discussion

There is evidence to suggest that breast cancer metastases are initiated by stem-like cells [11]. In the current study, we investigated breast CSC activity and metastasis using prospectively collected early and metastatic patient-derived samples *in vitro* and *in vivo*. We established that cells derived from metastatic fluids possessed an increased capacity to form both mammospheres *in vitro* and tumours *in vivo* compared to early breast cancer samples. Furthermore, we demonstrated that samples which metastasised to the lung in mice were those which possessed significantly higher mammosphere-forming efficiency *in vitro*.

To our knowledge, this is the largest prospective study of patient-derived tumour mammosphere formation. We compared mammosphere formation to clinical parameters across the whole patient cohort and found no correlation of mammosphere formation to clinical parameters such as tumour grade, molecular subtype or Nottingham Prognostic Index (NPI, derived from tumour size, number of involved lymph nodes and grade). However, higher mammosphere forming efficiency was observed in patients with ER-negative metastatic disease. This supports previous work where expression of the cancer stem cell marker ALDH1 has been shown to be more common in ER- tumours [13, 14]. *In vivo* tumour initiation capability did not correspond to the tumour characteristics of tumour grade, molecular subtype, hormone receptor status or NPI. The relationship between tumour characteristics and *in vivo* engraftment is controversial, with some studies showing hormone receptor status of the primary tumour to predict engraftment [15–18], and others showing no relationship [19, 20]. Our data adds to those studies suggesting that hormone receptor status does not predict engraftment *in vivo*.

Throughout this study we have utilised mammosphere culture to assess CSC activity *in vitro*. It should be noted that there are limitations to this technique. Aggregation of cells can occur, leading to a misinterpretation of results. To minimise cellular aggregation, we have fully optimised our protocols with regard to seeding density, culture time and culture conditions [12]. Further, we have previously established that mammospheres can be generated from a single cell [21], and that when single mammospheres are disaggregated and re-plated at one cell per well, only one mammosphere will form [7, 8]. These results demonstrate that the mammospheres reported in our study are not a result of cellular aggregation. Despite limitations, the mammosphere assay provides the advantage that it can be performed on a small number of isolated cells. Given the low yields generated from some of our early breast cancer samples (median cell number isolated; 35,000), this assay allowed us to gain an *in vitro* measure of CSC activity in a far larger number of samples than would have been achievable using FACS-based assays.

We did not observe a correlation between mammosphere formation *in vitro* and tumour initiation *in vivo*, our two measures of CSC activity. These have been previously shown to correlate in samples taken from metastatic breast cancer patients [22]. However, although tumour-propagating ability can reflect sphere-forming capacity, they do not always correlate. This is likely to be related to differences in environmental factors *in vitro* and *in vivo*. In fact, we previously reported a strong positive correlation between mammosphere formation and tumour initiation in limiting dilution assays once PDX models are established [23].

Patient samples derived from metastases had increased CSC activity compared to early tumours both as mammospheres *in vitro* and by initiating tumours *in vivo*. These results contrast to one previous study, which reported no difference between tumour initiation ability between early and metastatic samples [19], but support recent work demonstrating that recurrent tumours have a higher engraftment rate *in vivo* [18]. The increased CSC activity observed in metastatic samples suggests that CSCs are those which are able to survive conventional treatment and become metastatic in breast cancer patients. This supports results of two previous studies. Firstly it has been demonstrated that high expression of CD44+/CD24- CSCs in tumours favours distant metastases [24], and secondly patient treatment with chemotherapy has been shown to increase CSC activity (as assessed by CD44+/CD24- expression and mammosphere forming efficiency) in patient-derived tumours [25]. As all of the patients in our metastatic cohort had been exposed to chemotherapy, this may provide an explanation for the increase in CSC activity in this group. It is unknown if chemotherapy promotes the expansion of CSCs, or if CSCs are selected by chemotherapy based on their intrinsic drug resistance. A further hypothesis is that metastatic niche selectively supports the growth of cancer stem cells resulting in an expansion of their numbers in the metastatic setting. Although the mechanism remains unclear, our findings emphasise the importance of developing anti-CSC treatments to prevent and treat metastatic disease.

In our study, we prospectively derived 20 stable breast cancer patient-derived xenograft (PDX) models from 144 implanted tumours. Importantly, 11 of 20 were derived from ER positive tumours, a subtype which has historically been more difficult to successfully engraft [26]. Where we assessed spontaneous metastases in the models that formed tumours in the first generation, 14/34 (41 %) developed lung metastases, similar to previously published studies where 38/70 (54 %) PDX models were reported to develop lung metastases [15, 16, 19, 20, 27].

We are the first to assess metastatic capability in PDX models in relation to CSC frequency, and show that tumours which metastasise to the lung are those which have significantly higher CSC activity *in vitro*. Supporting this finding, a recent study demonstrated that single cells which metastasise in PDX models possess a gene signature similar to stem cells [11]. A limitation of studying metastasis in PDX models is that the frequency and sites of metastases in PDX models may differ from the patient [28]. Follow up data at 5 or more years post diagnosis will be important to help determine if metastases in our models correspond to that in the patients from which they were generated. Independent of this, these models will provide a useful biological resource for the breast cancer community, and will now be used as a platform to test new breast cancer anti-metastasis therapies.

In summary, both *in vitro* and *in vivo* CSC activities are increased in metastatic samples, and CSC activity *in vitro* predicts metastasis *in vivo*. These results suggest that breast CSC activity may predict for poor outcome tumours and that anti-CSC treatment should be utilised in the prevention and treatment of breast metastasis. Further validation of these results is now required, both in larger patient/PDX cohorts and by assessing patient follow-up data.

## Electronic supplementary material

Supplementary Table 1 **Summary of PDX models which spontaneously metastasise to the lung.** 14 PDX models demonstrated spontaneous lung metastases (8 EBC, 6 MBC). The number of mice assessed for metastases and the number of mice where metastases were present is shown. NA; information not available (PPTX 107 kb)

Supplementary Fig 1 **Sample processing of patient-derived samples**. Early breast cancers were removed at surgery and metastatic breast cancers were collected by percutaneous pleural effusion and ascitic fluid aspiration (a). Patients were either consented as “basic consent” or “animal consent”. Samples with basic consent were processed to give a single cell suspension and plated into the low adherence mammosphere assay. Mammospheres over ≥50 μm were counted at 7 days post plating (b). Samples with animal consent were additionally implanted into NSG mice, either as whole fragments (early breast cancers) or cell suspensions (metastatic breast cancers), and monitored for growth over 200 days. Mice where samples reached 1.3cm^3^ were culled and tumours were implanted into further generations of mice (c) (PPTX 487 kb)

Supplementary Fig 2 **Mammosphere formation related to clinical parameters and** ***in vivo*** **tumour initiation.** The relationship between mammosphere formation and clinical parameters was assessed in early and metastatic breast cancers. The oestrogen receptor (ER) status of the primary tumour did not predict mammosphere formation in early breast cancers (a), however in patients with metastatic disease those with ER negative tumours formed more mammospheres than patients with ER positive disease (p=0.0187) (b). Mammosphere formation was unrelated to molecular subtype (c, d) or grade (e, f) in either early or metastatic breast cancer, and was unrelated to Nottingham Prognostic index in early breast cancer (g). Mammosphere formation did not predict tumour growth *in vivo* in early breast cancers (h). Quick score positive for ER = >4. Nottingham prognostic index cut off poor ≥3.4. Data are represented as mean ± SEM. *p<0.05. (PPTX 159 kb)
