# Supplementary figures and images for: Patient-derived Mammosphere and Xenograft Tumour Initiation Correlates with Progression to Metastasis
Source: J Mammary Gland Biol Neoplasia. 2016 Sep 28;21(3):99–109. doi: 10.1007/s10911-016-9361-8 (PMC5159442; doi:10.1007/s10911-016-9361-8)

## Slide 1
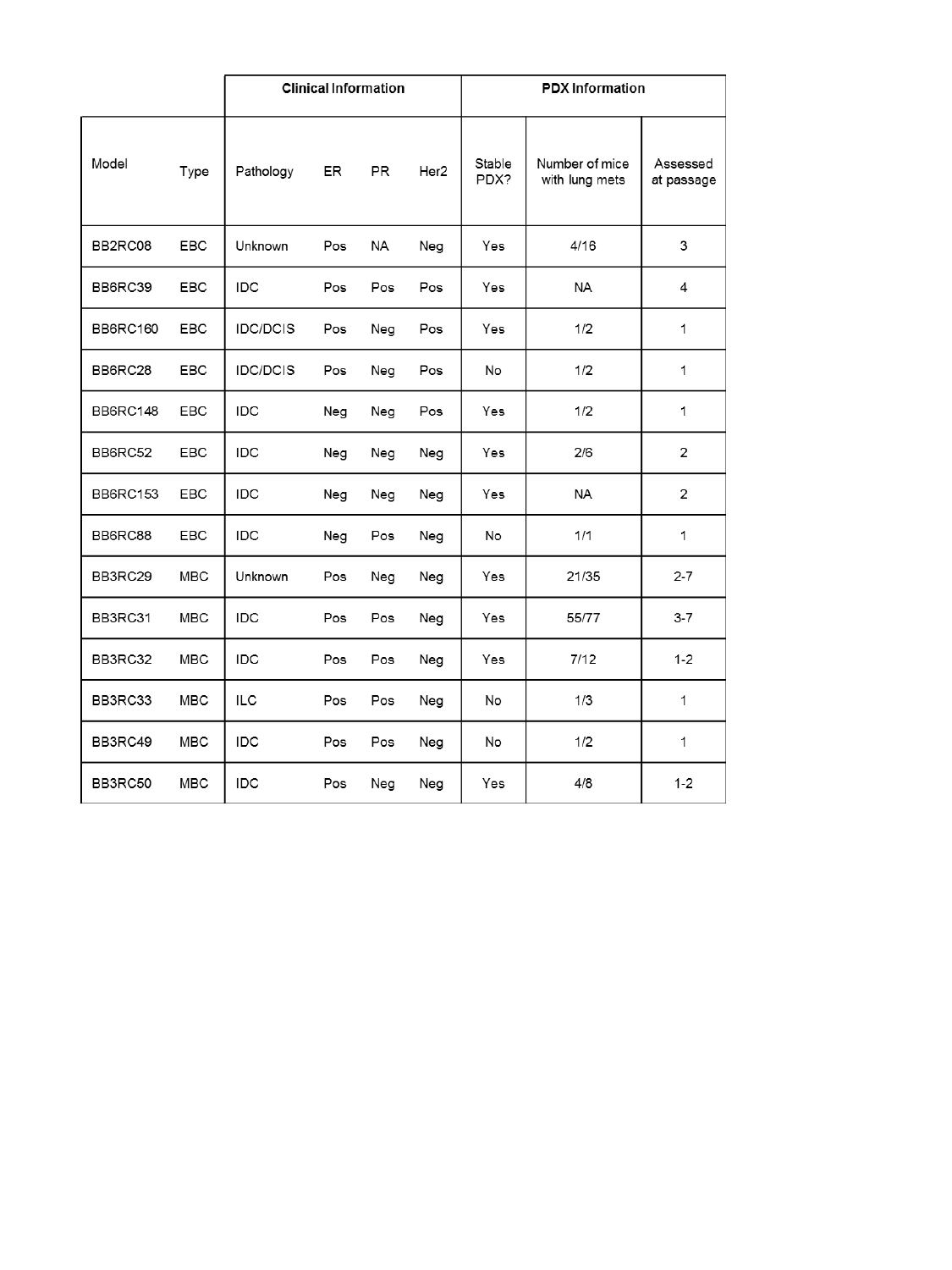

Supplement: Supplementary file 1 — Summary of PDX models which spontaneously metastasise to the lung. 14 PDX models demonstrated spontaneous lung metastases (8 EBC, 6 MBC). The number of mice assessed for metastases and the number of mice where metastases were present is shown. NA; information not available (PPTX 107 kb) [file 10911_2016_9361_MOESM1_ESM.pptx]

## Slide 1
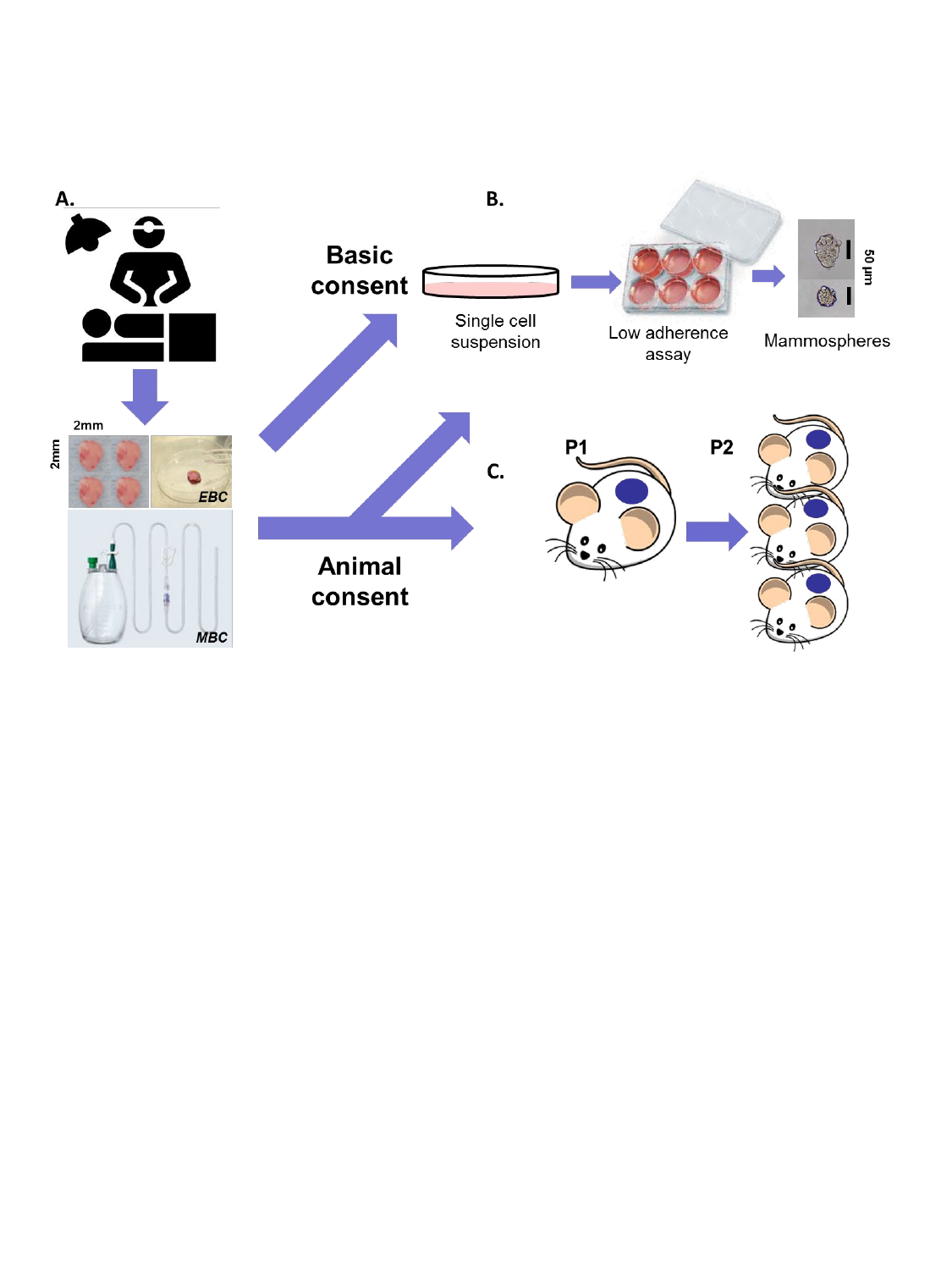

Supplement: Supplementary file 2 — Sample processing of patient-derived samples. Early breast cancers were removed at surgery and metastatic breast cancers were collected by percutaneous pleural effusion and ascitic fluid aspiration (a). Patients were either consented as “basic consent” or “animal consent”. Samples with basic consent were processed to give a single cell suspension and plated into the low adherence mammosphere assay. Mammospheres over ≥50 μm were counted at 7 days post plating (b). Samples with animal consent were additionally implanted into NSG mice, either as whole fragments (early breast cancers) or cell suspensions (metastatic breast cancers), and monitored for growth over 200 days. Mice where samples reached 1.3cm3 were culled and tumours were implanted into further generations of mice (c) (PPTX 487 kb) [file 10911_2016_9361_MOESM2_ESM.pptx]

## Slide 1
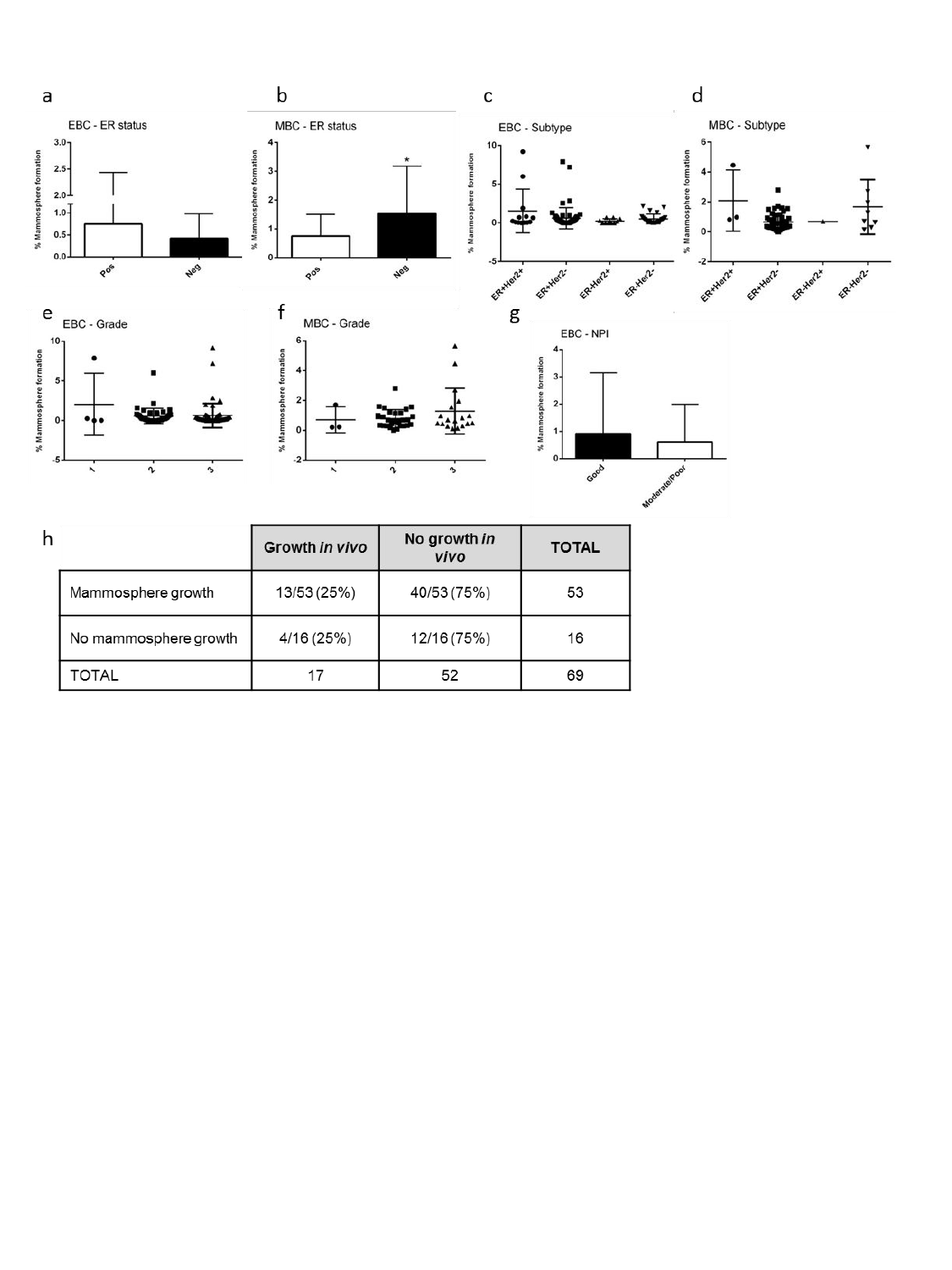

Supplement: Supplementary file 3 — Mammosphere formation related to clinical parameters and in vivo tumour initiation. The relationship between mammosphere formation and clinical parameters was assessed in early and metastatic breast cancers. The oestrogen receptor (ER) status of the primary tumour did not predict mammosphere formation in early breast cancers (a), however in patients with metastatic disease those with ER negative tumours formed more mammospheres than patients with ER positive disease (p=0.0187) (b). Mammosphere formation was unrelated to molecular subtype (c, d) or grade (e, f) in either early or metastatic breast cancer, and was unrelated to Nottingham Prognostic index in early breast cancer (g). Mammosphere formation did not predict tumour growth in vivo in early breast cancers (h). Quick score positive for ER = >4. Nottingham prognostic index cut off poor ≥3.4. Data are represented as mean ± SEM. *p<0.05. (PPTX 159 kb) [file 10911_2016_9361_MOESM3_ESM.pptx]
